# Mental Health Status and Associated Contributing Factors among Gay Men in China

**DOI:** 10.3390/ijerph15061065

**Published:** 2018-05-24

**Authors:** Xiaojun Liu, Dongdong Jiang, Xiangfan Chen, Anran Tan, Yitan Hou, Meikun He, Yuanan Lu, Zongfu Mao

**Affiliations:** 1 School of Health Sciences, Wuhan University, 115# Donghu Road, Wuhan 430071, China; xiaojunliu@whu.edu.cn (X.L.); 2017203050046@whu.edu.cn (D.J.); chenxiangfan@whu.edu.cn (X.C.); chloetar@whu.edu.cn (A.T.); houyitan@whu.edu.cn (Y.H.); 2017203050026@whu.edu.cn (M.H.); 2 Global Health Institute, Wuhan University, 115# Donghu Road, Wuhan 430071, China; yuanan@hawaii.edu; 3 Global Health Research Center, Duke Kunshan University, 8# Duke Road, Kunshan 215316, China; 4 Department of Public Health Sciences, University of Hawaii at Mānoa, 1960 East-West Road, Honolulu, HI 96822, USA

**Keywords:** Chinese gay men, mental health status, influencing factors, symptom checklist-90-R (SCL-90-R)

## Abstract

Chinese gay men are preferentially vulnerable to mental health problems because of deep-rooted, traditional social influence that overemphasizes heterosexual marriage, fertility, and filial piety. A cross-sectional survey was conducted from November to December 2017 using the Chinese version of the Symptom Checklist-90-R (SCL-90-R) to assess the status of, and factors associated with the mental health of Chinese gay men. Unadjusted associations between demographic factors and the total score of SCL-90-R were examined using *t*/*F* tests or person correlation analysis. The main factors that were most predictive of the aggregate score of SCL-90-R were identified by multiple linear regressions. A total of 367 gay men participated in this survey with an average score of SCL-90-R of 180.78 ± 79.58. The scores of seven dimensions (OCS, INTS, DEPR, ANX, HOS, PHOA, PARI) for Chinese gay men were found to be significantly higher than the national norm (all *p* < 0.001). Age (B = −1.088, SE = 0.478, *p* = 0.023), educational level (B = −14.053, SE = 5.270, *p* = 0.008), and degree of coming out publicly (B = −23.750, SE = 4.690, *p* < 0.001) were protective factors for participants’ mental health status. A gay man who is the only child in his family was more likely to obtain a higher total score of SCL-90-R in China (B = 59.321, SE = 7.798, *p* < 0.001). Our study reveals the worrying mental health status of Chinese gay men. Shifts in familial, governmental, and societal normas are suggested to improve the current social acceptance towards sexual minority men, as well as to reduce detrimental health effects.

## 1. Introduction

Sexual minority individuals are subject to more misunderstanding, prejudice, discrimination, and even insult compared with heterosexuals [1,2,3,4,5,6,7,8]. Perceptions and treatment of homosexuality in today’s society has been improved over the past few decades, with the American Psychiatric Association removing homosexuality from the classification criteria for the diagnosis of mental diseases in 1973 [9]. Understanding of sexual minority individuals has increased, which has led to more public acceptance, especially among younger, and more educated individuals [10,11,12]. As a result, countries or regions have begun to legally allow same-sex marriage, such as the Netherlands, Canada, and Taiwan [13]. Nevertheless, sexual minorities are more frequently misunderstood, mistreated, and experience higher levels of stress than their sexual majority counterparts, which seriously affects quality of life, mental health, and increases the risk of mental illness [2,5,6,7,14,15,16,17].

Current studies on sexual minorities are mainly carried out in Western countries, with research primarily centering on the causes of sexual orientation, social life and communication, sexual behaviors, and sexual health [1,2,3,4,5,6,7,8,9,10,11,12,13,14,15,16,17]. Notably, many studies have focused on men who have sex with men (MSM) and sexually transmitted diseases, such as HIV/AIDS. However, in recent years, the research direction towards homosexuality has shifted in Western countries; researchers have increasingly paid more attention to the mental health of MSM, including conducting studies on the effect of social negative attitudes towards the mental health of gay people [4,5,6,7,17,18,19,20,21,22,23,24,25,26,27]. In China, related studies are very limited since research is still primarily centered on the sexual health of MSM, especially sexual behaviors, sexually transmitted diseases, and HIV infections [28,29,30,31]. There are also several studies focused on the stigmatization of homosexuality [21,22,23]. However, the mental health of Chinese gay men and related influencing factors remains a blank spot for research.

China has always been deeply affected by the traditional mainstream thoughts of “treating woman as inferior to men”, “men take responsibility for carrying on the family line”, and “no descendants is the greatest unfilial crime”. However, gay men may not fit into these traditional beliefs of the Chinese man’s role. There are still common misconceptions and rejections of gay men in China’s society; a study conducted in three Asian cities–Hanoi, Shanghai, and Taipei–showed a low percentage of adolescents and young adults who hold a positive view of homosexuality [24]. Chinese gay men face stigma and discrimination in various domains of social life, such as in the lack of policy, legislation, and positive endorsement by governmental and socio-political organizations [22]. A study had confirmed that social status and relationships, the value of family, perceptions of immorality and abnormality, and gender stereotypes of masculinity are related to stigmatization of homosexuality in China [23]. Chinese gay men are under tremendous pressure and some of them are even forced to marry women, which is often reported by the media [25]. The public attitude towards gay men may negatively affect mental health, causing frustration, distress, and depression.

However, this important issue has not been obtained adequate attention in China, and there are few relevant studies conducted to focus on the Chinese gay men, especially their mental health. Therefore, based on the current Chinese social and cultural backgrounds, the present study is conducted to serve as the first study in China by employing the Chinese version Symptom Checklist- 90-R (SCL-90-R). The main aim of this study is to understand the current life, work, mental health, and social status among Chinese gay men and to analyze the factors affecting Chinese gay men’s mental health status.

## 2. Materials and Methods

### 2.1. Research Design and Setting

The present study is a cross-sectional survey study with data collected from both online and offline sources. The online survey was launched between November and December 2017 for a total of six weeks, to increase the diversity of participants. Participants were recruited exclusively through online methods by using gay dating apps (Blued, GayPark, Tantan, etc.) to send the questionnaire links, and shared the survey links to related internet gay dating groups like QQ groups and WeChat groups. Only those who self-identified as gay were included in our survey. Potential participants were invited to complete the survey when they agreed with our online informed consent. An offline survey was conducted by research group members from the Chinese Center for Disease Control and Prevention (China CDC). Survey questionnaires were distributed to the recruited volunteers who self-identified as gay at China CDC’s several pilot sites. Participation in this study was totally voluntary and anonymous, with no incentives provided to the responders.

### 2.2. Measures

#### 2.2.1. Demographic Information

The questionnaire captured social demographic characteristics of the survey participants, including participants’ age, marital status, education level, household registration, occupation, personal monthly income, average annual household income, sexual orientation disclosure status, and so on. In this study, we asked the participants to self-identify their sexual role preference with the option of top, bottom, or both. We also asked if the participants are the only child in the family with the option of yes or no (Table 1).

#### 2.2.2. The Chinese Version SCL-90-R

The present study employed the Chinese version SCL-90-R to assess the mental disorders and psychological health conditions of Chinese gay men. The SCL-90-R can evaluate a broad range of psychological problems and symptoms of psychopathology, including somatization (SOM), obsessive-compulsive symptoms (OCS), interpersonal sensitivity (INTS), depression (DEPR), anxiety (ANX), hostility (HOS), phobic anxiety (PHOA), paranoid ideation (PARI), and psychoticism (PSY). The SCL-90-R was verified as a popular and useful tool in diagnosing or measuring the progress and outcome of psychiatric and psychological treatments or for research purposes [32,33,34,35]. The Chinese version of SCL-90-R was introduced and developed by Wang Zhengyu in 1984 [34] and was confirmed to have good reliability and validity by some Chinese scholars [33,36]. A five-point scale, from 1 (not at all) to 5 (extremely), is used throughout the SCL-90-R. The average scores for each dimension are reported between 1 and 5, with a total score between 90 and 450; the higher the score of the SCL-90-R indicates greater risk for mental health issues.

### 2.3. Statistical Analysis

Statistical Package for the Social Sciences (SPSS) version 23.0 for Windows (SPSS Inc., Chicago, IL, USA) was employed to perform all data analyses, with a significance level of 0.05 (two-tailed). Frequencies and proportions were used to display the categorical variables, and metric variables were presented as mean and standard deviation. The average scores of all dimensions of Chinese version SCL-90-R in this study were compared to the reference range of China using *t*-tests. Unadjusted associations between demographic characteristics and the total score of SCL-90-R were examined using *t*/*F* tests or Pearson correlation analysis. The influencing factors associated with the aggregate score of Chinese version SCL-90-R were identified using multiple linear stepwise regression method. The multivariable model included all demographic variables, and model selection was automated.

### 2.4. Ethical Statements

This study was conducted in accordance with the Declaration of Helsinki. The data analysis was reviewed and exempted by the School of Health Science IRB of Wuhan University. The China Center for Disease Control and Prevention IRB also approved this study (MS2017024), and offered us great help in the offline data collection process.

## 3. Results

### 3.1. Descriptions of Sample Characteristics

A total of 367 Chinese gay men voluntarily participated in this survey study, averaging 27.9 years old (ranging between 15 and 62). Among these participants, 16.6% of them were married to women (*n* = 61), 51.5% (*n* = 189) received a college level education, and 43.9% (*n* = 161) were the only child of their families, and 47.1% (*n* = 173) came from rural areas. As shown in Table 1, 10.9% (*n* = 40) of these participants had no stable jobs, and over 50% had low personal monthly incomes (≤5000 CHY; *n* = 232); 67 (18.2%) self-identified as ‘top’–or the insertive/penetrating sexual partner–and 113 (30.8%) as ‘bottom’–the receptive sexual partner. It is noteworthy that only 4.4% of these Chinese gay men chose to be fully open about their sexual orientation, but the majority (56.9%) chose not to tell anyone. Details on the demographics of these participants are summarized in Table 1.

### 3.2. Assessment on Chinese Gay Men’s Present Situation of the Psychological Health

Table 2 records the Chinese gay men’s scores in nine dimensions of SCL-90-R, and it illustrates that the scores of seven dimensions (OCS, INTS, DEPR, ANX, HOS, PHOA, PARI) for gay men appear to be significantly higher than the Chinese national norm. No statistically significant difference was observed in the PSY domain (*t* = −0.66, *p* = 0.506) between the Chinese gay men’s practical score and national norm. The Chinese gay men’s total score for mental health was 180.78 ± 79.58.

### 3.3. Factors Affecting Chinese Gay Men’s Total Score of Mental Health

The Chinese gay men’s total score of SCL-90-R exhibited significant differences across different demographic characteristics including age (*rs* = −0.142, *p* = 0.007), marital status (*F* = 7.737, *p* = 0.001), educational level (*F* = 12.854, 0 < *p* < 0.001), occupation (*F* = 12.120, 0 < *p* < 0.001), personal monthly income (*F* = 17.067, 0 < *p* < 0.001), average annual household income (*F* = 14.525, 0 < *p* < 0.001), sexual role preference (*F* = 19.067, 0 < *p* < 0.001), degree of coming out publicly (*F* = 43.926, 0 < *p* < 0.001), and whether the only child in the family (*t* = −11.986, 0 < *p* < 0.001) (Table 3). The results of pairwise comparison illustrates that these divorced or widowed gay men obtained lowest score of SCL-90-R in China, followed by the more-educated men, indicating a more healthy psychological condition among these individuals. Being the only child in the family scored higher on test of SCL-90-R self-rating scale, which indicates they are in the most at-risk mental health state. These self-employed gay men in China seemed to be in the better psychological health status, for they showed the lowest score of SCL-90-R in this study. Moreover, the lower the income of Chinese gay men, the higher the score. These self-identified their sexual role preference as ‘bottom’ showed the highest score of SCL-90-R. Those who were completely confidential about their sexual orientation also scored highest. The higher score of the SCL-90-R indicates higher risk for mental health.

In order to identify the main factors that were most predictive of the Chinese gay men’s total score for mental health, multivariate linear regression analyses were conducted with an α = 0.05 entry standard. The results from the final multivariate linear regression analyses are shown in Table 4 after eliminating confounding factors. Five variables remained including marital status, age, educational level, degree of coming out publicly, and being the only child in his family. Analysis indicated that age (B = −1.088, SE = 0.478, *p* = 0.023), educational level (B = −14.053, SE = 5.270, *p* = 0.008), and degree of coming out publicly (B = −23.750, SE = 4.690, *p* < 0.001) are protective factors. A gay man who is the only child in his family is more likely to obtain a higher total score of SCL-90-R in China (B = 59.321, SE = 7.798, *p* < 0.001), indicating higher risk and more serious psychological problems and associated psychopathology symptoms. It is interesting to note that those divorced or widowed individuals (B = −13.653, SE = 11.489, *p* = 0.003) tend to receive lower total score of SCL-90- R as compared with the unmarried ones. However, there was no statistically significant difference (B = −7.732, SE = 10.964, *p* = 0.481) between married and unmarried gay men.

## 4. Discussion

In today’s China, the social environment has become more diversified and open with the development of economic globalization, international trade, research, education, and global cultural exchanges. However, on the other hand, many Chinese people—especially the older generation—are still hostile to sexual minorities due to the deep-rooted traditional moral influence that overemphasizes marriage, fertility, and filial piety [37,38,39]. Males with these traditional morals and religious beliefs are considered to be responsible for maintaining the family bloodline. For this reason, gay men in China may come under even greater pressure of marriage and fertility from parents or their family, and this pressure may also magnified by the traditional social morality and concepts. Consequently, Chinese gay men may be preferentially vulnerable to mental health problems and psychopathology symptoms, such as depression, anxiety, hostility, and so on [37,38,39,40,41].

In this cross-sectional study, we found that the SCL-90-R score for eight dimensions obtained from Chinese gay men was completely different from the Chinese national norm. Specifically, Chinese gay men were more likely to experience SCL-90-R mental health problems in OCS, INTS, DEPR, ANX, HOS, PHOA and PARI, for the score of these seven dimensions were statistical significantly higher than the Chinese national norm. These results indicate worrisome circumstances with regards to psychological issues and symptoms of psychopathology among the Chinese gay men. Körner et al. and Cohen et al. noted mental health problems and symptoms of psychopathology are likely manifest in gay men [42,43]. We speculate that this may be due to the insufficient social acceptance of gay men in China, limited public knowledge and bias relating to gay men may also play a role. Therefore, Chinese society needs to be more open, pluralistic, and friendly to gay individuals, and give them more understanding, support, and humanistic care. On the other hand, gay related stigma—referring to the prejudice, ignorance, insult, and discrimination towards to sexual minorities—should be controlled as effectively as possible.

Not surprisingly, this survey study also revealed that the participants’ somatization score was lower than the Chinese national norm. However, this result does not state that Chinese gay men are physically healthier due to the fact that the respondents in this study were mainly the young men (average age 27.9). Furthermore, no statistically significant difference between gay men and the general population was detected in the psychoticism dimension.

Consistent with the findings from previous studies focused on other populations, we found that Chinese gay men’s mental health status was also significantly associated with their age. Specifically, an older gay man was less likely to experience psychological health problems compared to younger gay men. This is understandable because people will become more experienced, mature, and accountable in coping with life and work-related stress as they grow older. Some existing studies have also demonstrated that the period of onset for mental illness symptoms often begins in adolescence and continues to young adulthood [43,44,45,46,47]. Therefore, young gay men can be a subgroup especially vulnerable to psychological problems which can even lead to suicide in some individuals. Hence, internal understanding and support from families are extremely important for gay individuals, especially younger men. Moreover, we also demonstrated that educational attainment has a positive effect on Chinese gay men’s mental health, indicating that more-educated individuals were less likely to suffer from mental health problems and symptoms of psychopathology.

To the best of our knowledge, the present study for the first time, showed that Chinese gay men who are the only child in their families were especially vulnerable to suffering mental health problems as compared to those gay men having siblings. This might be due to the deep-rooted influence of some traditional morals and religious beliefs that overemphasize the marriage, fertility, and filial piety. Ryan et al. revealed that gay men could experience prejudice and rejection from their family when disclosing their sexual orientation, which in turn can heighten gay men’s risk for mental health problems [48]. Considering the impacts of China’s one-child policy that has been implemented for over 30 years, it is important for the government to take relevant actions to give sexual minorities the same rights as the general population, increase social acceptance of sexual minorities, and reduce social inequality, for example, to legalize gay marriage [49].

In addition, our study indicated that less than 5% of the Chinese gay men chose to fully disclose their sexual orientation in this study, but 56.9% chose not to tell anyone, not even their own family members nor best friends. Interestingly, however, our study pointed out that there is a cross-sectional association between sexual orientation disclosure status and mental health status among Chinese gay men; specifically, if a gay man was more open on his sexual orientation, it could reduce his own risk on mental health problems. Thus, Chinese society needs to be more free, inclusive, and open to encourage gay men to disclose sexual orientation freely without fear or worry. Findings from this study also revealed that marriage with a woman is not a protective factor for Chinese gay men’s mental health and showed that those divorced or widowed participants tend to have the best mental health conditions. It is important to note that the current presence of a phenomenon of ‘fraudulent marriage’ in China today that the homosexuality was ‘forced’ by a variety of reasons including pressure from family and society, to have a heterosexual marriage by hiding their true sexual orientation, which is often reported by the media. Therefore, related actions need to be taken immediately to improve society’s acceptance of homosexuality as suggested above, and both the government and the community should engage in addressing this worrying issue actively.

## 5. Limitations

Given the fact that there are a very few studies conducted in China to investigate and explore mental health conditions of sexual minorities, and limited literature is available for reference. As China’s first research that focused on this special population, the following limitations of this study should be noted: first, our cross-sectional study design only reflects the current mental health status of Chinese gay men, and limits understanding of causality and the nature of relationships between mental health status and the causes of psychological problems, especially the dynamically changing social factors and ecological factors, such as gay men in heterosexual marriages. Second, non- response bias was not assessed, as only those agreeing to participate were included in this study and analysis. Moreover, we adopted non-random sampling, which can lead to a certain bias. Taing the online survey as an example, only those who utilize the Internet or use gay social networking dating apps were involved in the survey. Additionally, most of the participants are young, as the average age of this study is 27.9, hence, the results may not necessarily generalizable to older gay men. Fourth, although the SCL-90-R scores obtained from Chinese gay men in our study were statistically significantly higher than the Chinese national norm, it may be still underestimated, because we received a lot of rejections and even rejections with misunderstandings during the investigation. The volunteers who participated in this study maybe more outgoing and easygoing, and they tend to be in better mental health status. Finally, the present study was conducted with a very limited number of participants. Thus, future work should be conducted by considering all the limitations mentioned above.

## 6. Conclusions

Gay men are a vulnerable subgroup with psychological health problems and psychopathology symptoms. The current study reveals that Chinese gay men are more likely to experience higher SCL-90-R mental health scores in OCS, INTS, DEPR, ANX, HOS, PHOA, and PARI. Our study also shows older age and higher educational level are protective factors for gay men’s mental health status. A particular concern found in this study is that those Chinese gay men who are the only child in their families are especially vulnerable to mental health problems. This study also shows that most gay men in China prefer to keep their sexual orientation confidential, with some even engaging in heterosexual marriage. However, neither a heterosexual marriage nor concealing one’s sexual orientation can reduce the risk of, and/or prevent, Chinese gay men from mental health problems. Therefore, a combined effort from families, government organizations, and community members should be taken immediately in order to improve the social acceptance towards homosexuality, and make China’s social environment sufficiently open and truly diverse. Findings from this study also call for more public understanding, humanistic care, and support for sexual minorities to help gay men decrease the detrimental effects on their mental health problems. Moreover, more active in-depth studies relevant to the mental health of sexual minorities and its influencing factors are desperately needed in China.

## Figures and Tables

**Table 1:** Demographic information of the survey participants (*n* = 367).

Categorical Variables		Frequency	Percentage (%)
Marital status	Unmarried	275	74.9
	In marriage	61	16.6
	Divorced/Widowed	31	8.5
Education level	Junior high school and lower	36	9.8
	High school	102	27.8
	College	189	51.5
	Post-graduate and higher	40	10.9
Being the only child in the family	No	206	56.1
	Yes	161	43.9
Household registration	Countryside	173	47.1
	City	194	52.9
Occupation	Temporary jobs	40	10.9
	Migrant rural workers	54	14.6
	Self-employed	67	18.3
	Company employees	67	18.3
	Government employees	40	10.9
	Others	99	27.0
Personal monthly income (CHY)	3000 and lower	140	38.1
	3001~5000	92	25.1
	5001~7000	62	16.9
	7001~9000	41	11.2
	9000 and higher	32	8.7
Average annual household income (CHY)	15,000 and lower	63	17.2
	15,001~30,000	63	17.2
	30,001~45,000	88	24.0
	45,001~60,000	81	22.0
	60,001~75,000	43	11.7
	75,001 and higher	29	7.9
Sexual role preference	Top	67	18.2
	Bottom	113	30.8
	Both	187	51.0
Sexual orientation disclosure status	Confidential	209	56.9
	Either families or best friends	92	25.1
	Families and best friends	50	13.6
	Full disclosure	16	4.4
**Metric variables**		**Mean**	**S.D** **.**
Age (years)		27.9	8.8

**Table 2:** Assessment results of each dimension of SCL-90-R.

Dimensions	Reference Range of China ($\bar{x}$ ± *S*)	Participants’ Score in This Study ($\bar{x}$ ± *S*)	*t*	*p*
SOM	1.48 ± 0.54	1.38 ± 0.56	−3.26	0.001
OCS	1.83 ± 0.64	2.18 ± 1.03	6.52	0 < *p* < 0.001
INTS	1.68 ± 0.65	2.32 ± 1.08	11.39	0 < *p* < 0.001
DEPR	1.70 ± 0.65	2.32 ± 1.14	10.52	0 < *p* < 0.001
ANX	1.55 ± 0.55	2.08 ± 1.03	9.78	0 < *p* < 0.001
HOS	1.64 ± 0.63	2.32 ± 1.07	12.27	0 < *p* < 0.001
PHOA	1.40 ± 0.50	2.10 ± 1.08	12.47	0 < *p* < 0.001
PARI	1.58 ± 0.63	2.15 ± 1.15	9.58	0 < *p* < 0.001
PSY	1.53 ± 0.56	1.50 ± 0.71	−0.66	0.506
Total score	-	180.78 ± 79.58	-	-

**Table 3:** Associations between demographic characteristics and the total score of SCL-90-R.

Demographic Variables of Respondents		Mean	S.D.	*t/F/r_s_*	*p*	Pairwise Comparison
Marital status	Unmarried (g 1)	190.56	80.00	7.737	0.001	g 1 > g 2 > g 3
	In marriage (g 2)	172.00	75.60			
	Divorced/Widowed (g 3)	135.00	64.98			
Educational level	Junior high school or lower (g 1)	235.19	78.06	12.854	0 < *p* < 0.001	g 1 > g 2 > g 3 > g 4
	High school (g 2)	203.07	84.68			
	College (g 3)	169.03	73.77			
	Post-graduate and higher (g 4)	148.88	58.72			
Being the only child in the family	No (g 1)	145.45	58.50	−11.986	0 < *p* < 0.001	g 2 > g 1
	Yes (g 2)	230.56	77.52			
Household registration	Countryside (g 1)	184.72	78.29	0.441	0.660	-
	City (g 2)	181.06	80.87			
Occupation	Temporary jobs (g 1)	216.33	81.48	12.120	0 < *p* < 0.001	g 1 > g 5 & g 6 > g 3, g 2 > g 4 & g 6 > g 3, g 4 > g 5 > g 3
	Migrant rural workers (g 2)	221.11	78.70			
	Self-employed (g 3)	133.46	49.60			
	Company employees (g 4)	194.22	77.30			
	Government employees (g 5)	152.77	66.22			
	Others (g 6)	186.09	82.67			
Personal monthly income (CHY)	3000 and lower (g 1)	182.94	82.35	17.067	0 < *p* < 0.001	g 2 > g 1 & g 3 > g 4 & g 5
	3001~5000 (g 2)	224.90	72.94			
	5001~7000 (g 3)	181.11	72.98			
	7001~9000 (g 4)	139.66	64.71			
	9000 and higher (g 5)	119.50	31.50			
Average annual household income (CHY)	15,000 and lower (g 1)	217.95	81.82	14.525	0 < *p* < 0.001	g 2 > g 1 > g 3 > g 4 & g 5 & g 6
	15,001~30,000 (g 2)	266.62	76.68			
	30,001~45,000 (g 3)	186.75	76.22			
	45,001~60,000 (g 4)	145.52	70.00			
	60,001~75,000 (g 5)	147.07	64.07			
	75,001 and higher (g 6)	156.17	53.49			
Sexual role preference	Top (g 1)	141.21	59.69	19.067	0 < *p* < 0.001	g 2 > g 3 > g 1
	Bottom (g 2)	212.83	71.15			
	Both (g 3)	179.52	83.47			
Sexual orientation disclosure status	Confidential (g 1)	217.61	77.18	43.926	0 < *p* < 0.001	g 1 > g 2 & g 3 & g 4, g 2 > g 3
	Only families/best friends (g 2)	147.25	59.40			
	Families and best friends (g 3)	117.58	34.18			
	Full disclosure (g 4)	135.94	74.41			
Age (years)		-	-	–0.142	0.007	-

**Table 4:** Predictors of factors associated with the total score of SCL-90-R among Chinese gay men.

Variables	Unstandardized Coefficients		Standardized Coefficients	*t*	*p*	95% CI for *B*	
	*B*	S.E	Beta			Lower	Upper
**Marital status**							
Unmarried (reference group)							
In marriage	−7.732	10.964	−0.034	−0.705	0.481	−29.294	13.831
Divorced/Widowed	−13.653	11.489	−0.124	−3.019	0.003	−22.726	−4.658
Being the only child in the family	59.321	7.798	0.370	7.607	0 < *p* < 0.001	43.985	74.657
Sexual orientation disclosure status	−23.750	4.690	−0.261	−5.064	0 < *p* < 0.001	−32.975	−14.526
Age (years)	−1.088	0.478	−0.121	−2.277	0.023	−2.028	−0.148
Educational level	−14.053	5.270	−0.142	−2.667	0.008	−24.417	−3.689
*F* = 19.044, *R*^2^ = 0.371, 0 < *p* < 0.001							

Note: B = Coefficient; S.E = Standard error.
